# Supplementary figures and images for: Armadillo repeat-containing protein 1 is a dual localization protein associated with mitochondrial intermembrane space bridging complex
Source: PLoS One. 2019 Oct 23;14(10):e0218303. doi: 10.1371/journal.pone.0218303 (PMC6808451; doi:10.1371/journal.pone.0218303)

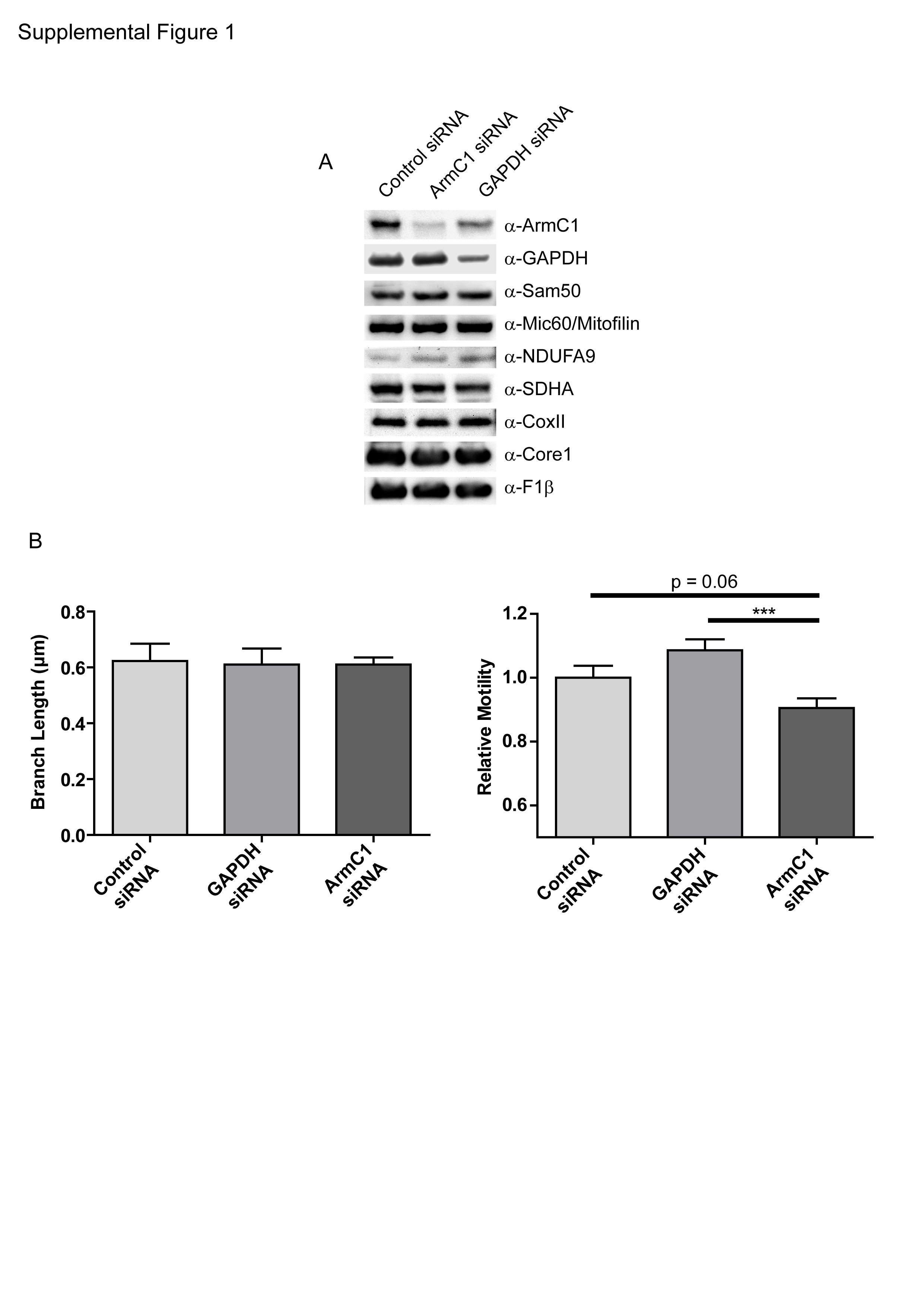

Supplement: S1 Fig — (A) HeLa cells were transfected with control, GAPDH and ArmC1 siRNA pools and 72 h post transfection analyzed by western blot, using designated antibodies. (B) HeLa cell line expressing mitochondrial matrix targeted GFP (CoxVa presequence-GFP) was transfected with control, GAPDH and ArmC1 siRNA pools and 72 h post transfection stained with SIR-tubulin and imaged once per minute by confocal microscope for 10–15 minutes. Mitochondrial average length was analyzed from one frame at the beginning of imaging. Mitochondrial motility between the frames was measured and normalized to control siRNA pool results. The graph represents mean values ±SD. Significance was calculated using Student’s t test.: ***—p≤0.001. Sam50, sorting and assembly machinery 50, Tom, translocase of the outer mitochondrial membrane, NDUFA9, NADH dehydrogenase [ubiquinone] 1 alpha subcomplex subunit 9, SDHA, Succinate dehydrogenase complex subunit A, Cox, cytochrome oxidase, F1β, ATP synthase subunit beta, GAPDH, Glyceraldehyde 3-phosphate dehydrogenase. (TIF) [file pone.0218303.s001.tif]
